# Historical milestone in 42 years of viral sequencing—Impetus for a community-driven sequencing of global priority pathogens

**DOI:** 10.3389/fmicb.2022.1020148

**Published:** 2022-11-04

**Authors:** Li Chuin Chong, Asif M. Khan

**Affiliations:** ^1^Beykoz Institute of Life Sciences and Biotechnology, Bezmialem Vakif University, Istanbul, Turkey; ^2^School of Data Sciences, Perdana University, Kuala Lumpur, Malaysia

**Keywords:** community, virus, SARS-CoV-2, health policy, monkeypox, sequencing, pathogen

## Introduction

Sequence data are critical for the design of effective intervention (vaccines, drugs, and diagnostics) and surveillance strategies against pathogens. This need is more than ever exemplified by the ongoing global scientific effort against the COVID-19 pandemic, where the resulting sequence data of the disease agent, severe acute respiratory syndrome coronavirus 2 (SARS-CoV-2) is currently the largest ever for a given virus.

## Trend and historical milestone in coronavirus sequencing

As many as 13,376,049 genome sequences, translating to 351,835,857 protein records (as of 4 October 2022) have been deposited to the specialist GISAID EpiCoV™ repository (Khare et al., 2021), the single largest open-access platform for SARS-CoV-2 sequence data, surpassing that of NCBI databases (NCBI Resource Coordinators, 2018), a long-standing primary sequence resource. GISAID, formally launched in 2008, has been able to encourage greater international sharing of viral sequence data through innovative policies and offerings that recognize the contributions and interests of data providers and users alike (Khare et al., 2021), starting with influenza virus. These include: (i) a trusted sharing mechanism framework that guarantees that data users will acknowledge the contributions of, and make efforts to collaborate with, data generators; (ii) a high-throughput submission portal; (iii) high quality data standards through review and curation in real-time and annotation by a global team of curators, prior to release, for all submitted data; (iv) enhancement of curated data with computed results; and (v) delivery for downstream analyses *via* customisable data feeds. Consequently, GISAID was in a position to serve as a critical blueprint for SARS-CoV-2 data deposition and real-time analyses, and is thus expected to be well-poised for future pandemic challenges.

**Figure 1:**
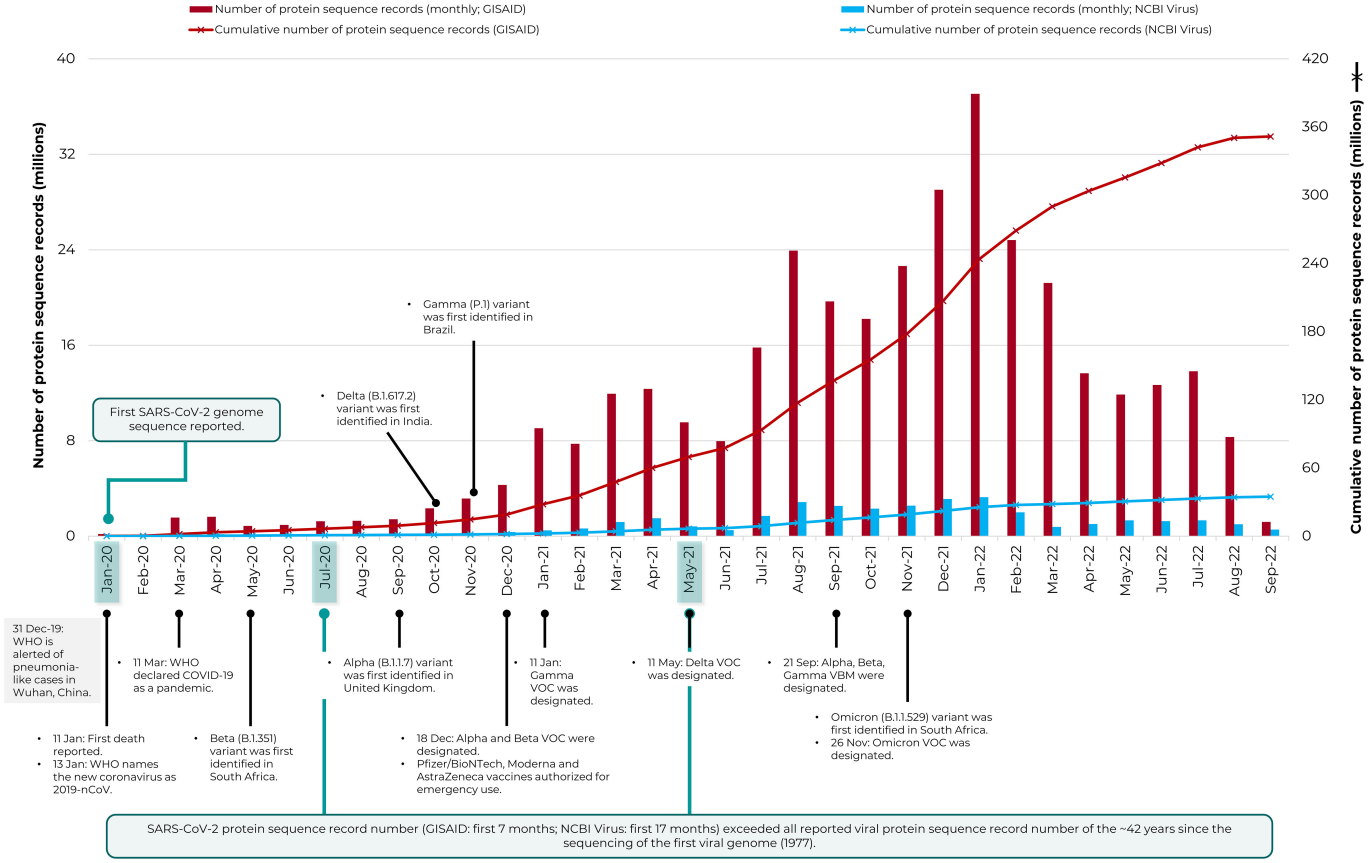
The rapid increase in protein sequence records of SARS-CoV-2 deposited in the specialist repository, GISAID EpiCoV™. Data is shown from January 2020 to September 2022. The NCBI Virus resource is included for comparison and the timeline of major COVID-19 pandemic events and sequencing milestones are also indicated (see Carvalho et al., 2021 and https://www.who.int/en/activities/tracking-SARS-CoV-2-variants/). VBM, Variants Being Monitored; VOC, Variants of Concern; WHO, World Health Organization.

Figure 1 depicts the number of protein sequence records available for SARS-CoV-2 in the specialist databases GISAID EpiCoV^TM^ and NCBI Virus (NCBI Resource Coordinators, 2018), with collection date starting from January 2020, before the onset of the pandemic to 33 months later, September 2022. The records in GISAID grew rapidly from ~0.02 million to ~351 million in less than three years, a number strikingly ~10-fold higher than NCBI Virus (up to ~35 million). This unprecedented, rapid collection of sequence data (GISAID) had edged past that of HIV-1 by March 2020, for which the greatest number of viral protein records had been reported, till before the pandemic (>1 million as of December 2019 in NCBI Virus; the species SARS-related coronavirus then just had only 4.4K records).

The concerted worldwide sequencing effort for SARS-CoV-2 has remarkably etched a historical milestone in virus sequencing, as the fastest and the single largest source of viral sequence data growth. The protein record data of SARS-CoV-2 (collected by first 7 months since January 2020 for GISAID or first 17 months for NCBI Virus) had exceeded the total number of protein records made available for all reported viruses since the 1977 sequencing of the first viral DNA genome, bacteriophage phi X 174 (or ΦX174) to December 2019, a period of ~42 years. There were then 5,948,418 protein records in NCBI Virus for all reported viruses (data not shown), released as of 31 December 2019. Likewise, the nucleotide record data of SARS-CoV-2 [collected by first 19 months for GISAID since January 2020 (https://gisaid.org/hcov-19-variants-dashboard) or first 22 months for NCBI Virus (data not shown)] had exceeded the number of such publicly available records of 42 years for all reported viruses (3,225,425 records in NCBI Virus as of 31 December 2019; data not shown). Notably, the GISAID data was largely of full-length genomes, whereas the prior data (of all viruses) was a mix of full-length genomes and partial sequences.

## Need for a community-driven sequencing of global priority pathogens

The coronavirus sequencing historical milestone hails the value of a worldwide, community-driven pathogen sequencing effort. It heralds a global, real-time surveillance and rapid intervention response against infectious diseases. Thus, there is a need to call for action to replicate this sequencing success as a model for other pathogens, starting with the long available list of global priority pathogens (NIAID, 2021) and perhaps with primacy given to viruses due to their smaller genome size. There is much that could be learnt from the experiences of GISAID (restricted open sharing), the International Nucleotide Sequence Database Collaboration (INSDC; promotes full open sharing), and other advocates of pathogen genomics (Black et al., 2020), where despite the differences in approach (Van Noorden, 2021), closing the global genome data gap (including metadata) for these pathogens is the collective priority (Mallapaty, 2022). Urgently needed is a global and an open platform for pathogen genomics, in the form of a consortium or an initiative and particularly involving scientific community members from affected countries, especially those of low- and middle-income countries (LMICs), with support from the vanguards of community-driven sequencing. This endeavor may possibly be sustained through assessed and voluntary funding contributions. Efforts that are already underway, such as the various national, international, and continent-wide pathogen sequencing and readiness consortiums/initiatives (Holmes et al., 2017; Makoni, 2020; NIAID, 2021; Harvard Medical School, 2022) can be adapted to expand globally (Illumina Inc., 2021). A bottom-up community-driven, open approach can be greatly complementary to the established efforts that are top-down and central, often initiated or led by large organizations, such as the CEPI (Brende et al., 2017) and Global Virome Project (Carroll et al., 2018), among others. The recipe for success is available, global collaborations are at an all-time high, and the pressing need is to rapidly model the successful coronavirus sequencing effort to all pathogens of priority first and of interest later. Granted that the model has its shortcomings, enabling global sequencing of clinical and environmental pathogen isolates is a critical first step toward the development of effective intervention and surveillance strategies.

## Discussion

COVID-19 remains a vivid reminder that a single isolated outbreak is capable of rapidly bringing the world to a grinding halt for a reasonable duration of time, crippling humanity with loss of lives and battered health systems. This happened in the scientifically advanced 21st century. Thus, the new normal of the post-COVID-19 era needs to maintain the momentum and advance the progress that has been made over the catastrophic sacrifice. Remaining blind spot gaps will need to be identified and addressed. A collective community action is a necessary catalyst that can help uniformly strengthen the flailing, porous fabric of our global preparedness. The recent surge of human monkeypox cases, 72,428 confirmed in 102 geographical locations where the disease is not typically reported (as of 14 October 2022) (CDC, 2022), and the concern of fitness selection as the virus variants find more hosts for continuous evolution, is a poignant reminder.

## Author contributions

AK developed the initial concepts for this paper and reviewed the writing. LC contributed to the data curation, formal analysis, visualization, and writing—original draft. Both authors approved the final version and had final responsibility for the decision to submit for publication.

## Funding

AK was supported by Perdana University, Malaysia, Bezmialem Vakif University, Turkey, and the Scientific and Technological Research Council of Turkey (TÜBITAK). This publication/paper has been produced benefiting from the 2232 International Fellowship for Outstanding Researchers Program of TÜBITAK (Project No: 118C314). However, the entire responsibility of the publication/paper belongs to the owner of the publication/paper. The financial support received from TÜBITAK does not mean that the content of the publication is approved in a scientific sense by TÜBITAK.

## Conflict of interest

The authors declare that the research was conducted in the absence of any commercial or financial relationships that could be construed as a potential conflict of interest.

## Publisher's note

All claims expressed in this article are solely those of the authors and do not necessarily represent those of their affiliated organizations, or those of the publisher, the editors and the reviewers. Any product that may be evaluated in this article, or claim that may be made by its manufacturer, is not guaranteed or endorsed by the publisher.

## Author disclaimer

The authors express that they are in no way connected to GISAID, besides being user of its various databases.
